# Effects of a ketogenic diet during pregnancy on embryonic growth in the mouse

**DOI:** 10.1186/1471-2393-13-109

**Published:** 2013-05-08

**Authors:** Dafna Sussman, Matthijs van Eede, Michael D Wong, Susan Lee Adamson, Mark Henkelman

**Affiliations:** 1 Department of Medical Biophysics, Faculty of Medicine, University of Toronto, Toronto, Canada; 2 Mouse Imaging Centre (MICe), The Hospital for Sick Children, Toronto, Canada; 3 Departments of Physiology, Obstetrics and Gynaecology, University of Toronto, Toronto, Canada; 4 Samuel Lunenfeld Research Institute, Mount Sinai Hospital, Toronto, Canada

**Keywords:** Ketogenic diet, Low-carbohydrate diet, Embryonic development, CD-1 mouse, Mouse imaging, Optical projection tomography, Magnetic resonance imaging

## Abstract

**Background:**

The increasing use of the ketogenic diet (KD), particularly by women of child-bearing age, raises a question about its suitability during gestation. To date, no studies have thoroughly investigated the direct implications of a gestational ketogenic diet on embryonic development.

**Methods:**

To fill this knowledge gap we imaged CD-1 mouse embryos whose mothers were fed either a Standard Diet (SD) or a KD 30 days prior to, as well as during gestation. Images were collected at embryonic days (E) 13.5 using Optical Projection Tomography (OPT) and at E17.5 using Magnetic Resonance Imaging (MRI).

**Results:**

An anatomical comparison of the SD and KD embryos revealed that at E13.5 the average KD embryo was volumetrically larger, possessed a relatively larger heart but smaller brain, and had a smaller pharynx, cervical spinal cord, hypothalamus, midbrain, and pons, compared with the average SD embryo. At E17.5 the KD embryo was found to be volumetrically smaller with a relatively smaller heart and thymus, but with enlarged cervical spine, thalamus, midbrain and pons.

**Conclusion:**

A ketogenic diet during gestation results in alterations in embryonic organ growth. Such alterations may be associated with organ dysfunction and potentially behavioral changes in postnatal life.

## Background

The Ketogenic Diet (KD) is a high fat, low carbohydrate, adequate protein diet, which has been gaining support as a lifestyle diet for weight maintenance [1] and body-building purposes in healthy adults [2]. Classically, the diet has been primarily used as a therapeutic measure for intractable pediatric epilepsy. However, due to its high efficacy and significant advantages over anti-epileptic medications, adult epileptic patients are recently adopting it as well. Its increased and prolonged use by adults, and specifically women, raises a question about its safety as a gestational diet. Gestational diets determine maternal nutrition, which is transmitted to the fetus through the placenta [3,4]. An insufficient or inappropriate gestational diet can cause permanent deleterious effects to maternal and fetal metabolism [5], leading to altered fetal physiology. The relationship between a gestational diet and fetal physiology is often referred to as “fetal developmental adaptation” [6-9]. The resulting altered fetal metabolism and physiology can affect organ development and function. In fact, such alterations in metabolism and physiology have been shown to predispose the fetus to multiple metabolic, endocrine, cardiovascular, mental, and cognitive disorders in adult life [10,11]. Thus, it is important to investigate whether a KD negatively impacts prenatal organ development to determine the safety and suitability of a KD during pregnancy.

Previous studies of gestational ketosis focused on ketosis as resulting from maternal malnutrition, prolonged fasting, or diabetes. These, however, are different than the stable ketosis that results from consumption of a ketogenic diet (KD) that is of adequate energy and nutrients.

The mouse is a convenient and commonly used model for studying the effects of environmental factors, including nutrition, on mammalian developmental. The mouse genome has been fully sequenced and was found to share a high degree of homology with the human genome. Such homology suggests that studying the physiological effects of a KD on mouse development, may shed light on the potentially-similar effects on human development. In the current study we compare the growth of mouse embryos on a KD with that of embryos on a Standard Diet (SD), and utilize three-dimensional (3D) imaging and quantification to test whether statistically significant diet-induced differences are evoked in prenatal organ and body growth. 3D imaging is a non-invasive, non-destructive technique that allows to identify patterns and analyze complex anatomical geometry within the mouse embryo. Optical Projection Tomography (OPT) was chosen for imaging the E13.5 embryos due to its fine spatial resolution of 20 *μm*. However, OPT is limited to spatial coverage of about (1-2cm) ^3^, as well as by tissue opacity which becomes significant beyond E14.5 [12]. Thus, to image the E17.5 embryos we utilized Magnetic Resonance Imaging (MRI) which provided a 56 *μm* isotropic resolution.

## Methods

Six-week-old CD-1 male and female mice were weight-matched and randomly assigned to either a control group (SD), or a study group (KD). Mice were fed their respective diets for a period of 30 days, and then naturally mated by setting up a single male with a single female from their respective group. The morning a copulation plug was observed was considered day 0.5 of gestation. The mated dams were allowed to reach embryonic (E) day E13.5 or E17.5 days post-conception, corresponding to the end of organogenesis and fetal growth, respectively [13]. At these time-points the pregnant dams were euthanized and several of their embryos were harvested for imaging. In both dietary groups, all embryos appeared viable, had no obvious malformations, and the selected ones were chosen arbitrarily.

### Diet

Both the Standard Diet (SD) and the Ketogenic Diet (KD) were manufactured by Harlan (Madison, WI). SD consisted of 5% fat, 76.1% carbohydrate, and 18.9% protein, wt/wt (Teklad diet no. TD.2918), whereas KD consisted of 67.4% fat, 0.6% carbohydrate, and 15.3% protein wt/wt, which is equivalent to a 4:1 ratio by weight of fat to combined protein and carbohydrate (Teklad diet no. TD.96355) [14]. The fatty-acid profiles of the SD / KD, respectively, are as follows: % Saturated Fatty Acids (SFA): 0.9 / 15.90, % Monounsaturated Fatty Acids (MUFA): 1.3 / 25.80, % Polyunsaturated Fatty Acids: 3.4 / 24.35. While the SD meets the nutritional needs of the pregnant mouse, the suitability of the KD during gestation has not been fully elucidated. During the initial 30-day feeding period, mice in the control group were kept on a standard rodent diet (SD), and mice in the study group were gradually introduced to the KD to facilitate gradual adaptation and metabolic shift from glycolysis to ketosis. The introduction of the KD was conducted over a 10-day period, after which the KD mice were given only the pure KD during the remaining 20 days in the feeding period. Preliminary studies showed that ketone values had stabilized by 4 weeks (not shown); hence, mating was performed at that time-point in all experiments.

### Animals

All animal procedures in this study were carried out in accordance with the Canadian Council on Animal Care, and approved by the Animal Care Committee of the Toronto Centre for Phenogenomics (TCP). During the 30-day feeding period, physiological and metabolic parameters were assessed in the first 20 animals per dietary group to determine whether the KD mice reached a stable-state ketosis within that time frame. Body weight, blood glucose and *β*−ketones (*β* − *hydroxybutyrate*) were measured weekly, using an Abbott “Precision Xtra” glucometer which required a relatively minute blood volume of 0.6-1.5 *μ*L/test [15] drawn from the tail vein. Cholesterol and triglycerides were tested at the end of the feeding period, and required a combined whole-blood volume of 190 *μ*L, collected from the saphenous vein. During gestation, weekly body weight and glucometer testing were measured in all mice. Metabolic data was statistically analyzed for changes over time and between dietary groups using Analysis of Variance (ANOVA) with a linear model [16].

### Imaging

All embryos were fixed for 24 hours in 4% para-formaldehyde. The E13.5 embryos were then embedded in 1% low melting temperature agar which was subsequently dehydrated in methanol and cleared in BABB (a 2:1 mixture of Benzyl-Benzoate:Benzyl-Alcohol) prior to being imaged with OPT using auto-fluorescence mode [17]. The fixed E17.5 embryos were immersed in PBS containing 4% Gadolinium (“ProHance” gadoteridol by Bracco Diagnostics) for 3 days, after which they were embedded in high melting temperature agar containing 4% Gd, and then imaged with MRI, using a T2-weighted, 3D fast-spin echo sequence.

**Figure 1:**
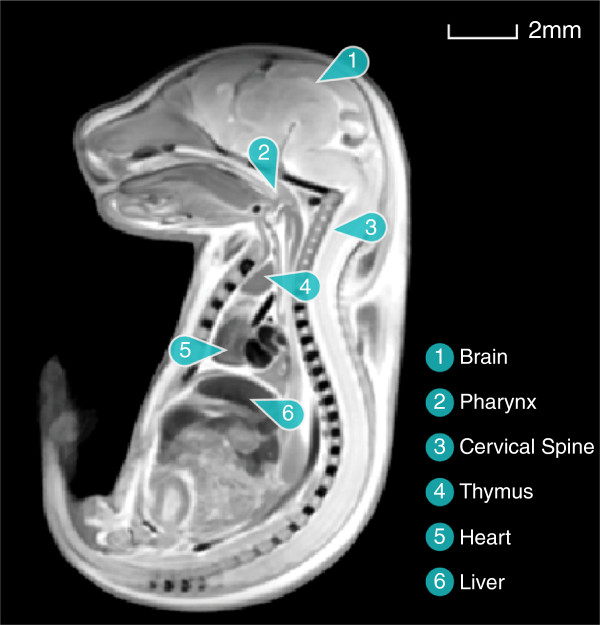
An image of an E17.5 mouse embryo with selected anatomical structures labeled.

An example of an embryo image is depicted in Figure 1, along with labeling of several anatomical structures.

### Image statistical analysis

For each of the two time-points, homologous points within all SD and KD images were detected, aligned with respect of one another, and saved within a common coordinate system. The overlapping images were then transformed into a single consensus average that accurately identified the anatomical correspondence between the different images. This transformation followed a multi-step process. The first step linearly aligned all images through a series of rotations, translations, scales, and shears. The second step used an iterative non-linear alignment procedure that created local deformations in each image that brought all images into exact correspondence. The collection of all linear and non-linear deformations across each image formed a *deformation field*, which was the transformation from each individual image of mouse embryo anatomy to the consensus average image. To reveal any local changes in each image with respect to the consensus average image, the determinant of each deformation field, known as the Jacobian, was computed for each image voxel (i.e. 3D pixel). These Jacobians were then analyzed statistically to determine whether the KD embryos significantly differed from the SD (control) ones in any particular 3D region. This analysis is referred to as a *deformation-based* analysis, and its results are reported as an imge overlaid with a red / blue color scale-bar indicating the significant t-statistics values of the corresponding voxels. To also investigate group-differences between volumes of specific organs, outlines of chosen organs were traced (segmented) in the average image, thereby isolating them in 3D space. To map these regions back to the original individual images and compute their original volumes, the Jacobian determinant was used: it was integrated, multiplied by the voxel size of the voxels residing within the organ [18]. This volume analysis will be referred to as an *organ-based* analysis. To correct for multiple comparisons in these analyses, a False Discovery Rate (FDR) method was utilized [19,20], and is reported along with the results.

## Results

### Metabolic status of pre-mated female mice

Average body weight and blood glucose of KD female mice did not statistically differ from those of SD female mice (p > 0.1) prior to mating. However, prior to mating, the serum ketone and cholesterol concentrations of KD female mice were significantly elevated, and their serum triglyceride concentrations were significantly lower compared with those of SD female mice (p < 0.05), as anticipated for the KD diet (Table 1).

**Table 1:** Body weight and blood metabolites of female SD and KD mice

		**Weight**	**Glucose**	**Ketone**	**Cholesterol**	**Triglyceride**	**Num. of**
		**[gr]**	**[mmol/L]**	**[mmol/L]**	**[mmol/L]**	**[mmol/L]**	**embryos**
Prior to	SD	29.5±0.5 (20)	6.4±0.3 (20)	0.25±0.02 (20)	3.4±0.2 (20)	2.1±0.2 (20)	-
Mating	KD	30.8±0.7 (20)	5.3±0.2 (20) *	1.94±0.15 (20) *	4.4±0.2 (17) *	1.8±0.1 (17) *	-
At E13.5	SD	45.0±1.3 (11)	7.0±0.5 (11)	0.22±0.04 (11)	2.8±0.1 (10)	2.8±0.2 (10)	12.9±0.6 (17)
	KD	45.4±1.2 (11)	6.6±0.5 (11)	1.57±0.23 (11) *	3.6±0.2 (11) *	5.1±1.1 (11) *	12.1±1.0 (12)
At E17.5	SD	67.1±1.7 (19)	5.9±0.3 (12)	0.27±0.04 (11)	2.0±0.1 (13)	2.7±0.3 (13)	10.9±1.2 (8)
	KD	58.9±1.1 (18) *	2.8±0.2 (11) *	3.96±0.30 (11) *	2.8±0.3 (12) *	7.8±1.8 (12) *	11.3±0.6 (6)
p-value	Diet	0.0094	< 0.0001	< 0.0001	< 0.0001	0.0569	–
	Time-pt	< 0.0001	0.0055	0.0001	< 0.0001	0.0011	–
	Interaction	0.0008	0.0411	0.0001	0.0273	0.0481	–

Values were measured just prior to mating, at E13.5 and at E17.5 (*Mean* ± *SEM* (Number of dams tested)). Statistically significant KD vs. SD p-values are indicated by a (*) for comparison at each time point.

### Embryonic imaging

**Figure 2:**
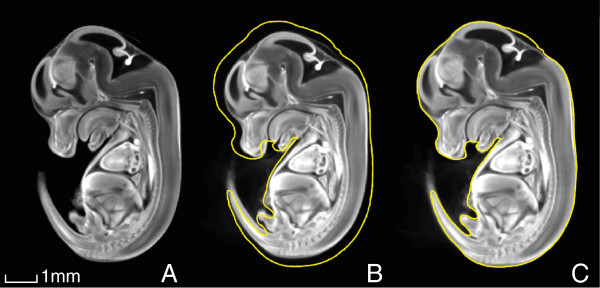
**Embryonic size difference at E13.5.** (**A**) A consensus average of all E13.5 embryos constructed using the SD and KD OPT embryo images (N = 59), followed by separate averages of (**B**) all SD (N = 27) and (**C**) all KD (N = 32) E13.5 embryos. The outline of the KD embryo is traced in yellow in (b) to illustrate the overall size difference compared with the SD embryo.

**Figure 3:**
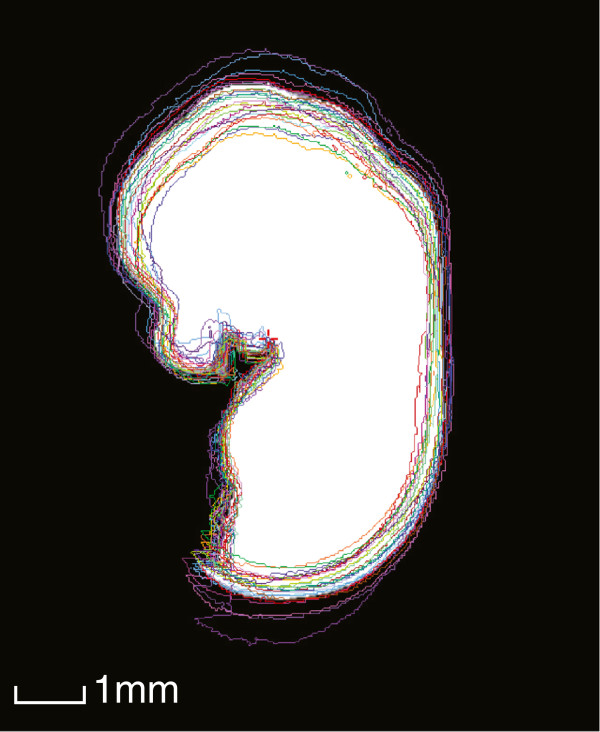
**An illustration of the animal-to-animal variation within the SD group at E13.5.** The coloured traces are outlines of all linearly-aligned individual SD images, and the white region is a mask of the final average image.

A significant size difference was observed during imaging of E13.5 KD and SD embryo, as depicted in Figure 2. This figure also depicts the final average embryo image, constructed using all SD and KD embryos (57 embryos in total), which retained its high resolution during registration. To assess the animal-to-animal variation among the SD mice, the outlines of all linearly-aligned SD images were overlaid on top of a white mask of the final E13.5 average image. This is depicted in Figure 3.

**Figure 4:**
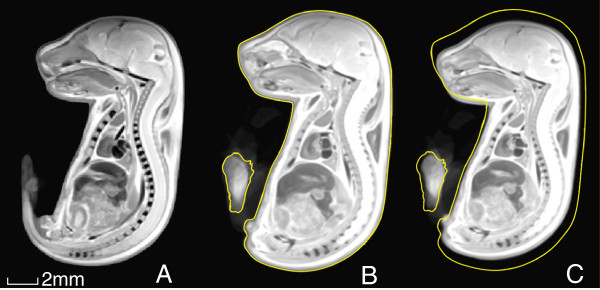
**Embryonic size difference at E17.5.** (**A**) A consensus average of all E17.5 embryos constructed using the SD and KD MRI embryo images (N = 20), followed by separate averages of (**B**) all SD (N = 10) and (**C**) all KD (N = 10) E17.5 embryos. The outline of the SD embryo is traced in yellow in (c) to illustrate the overall size difference compared with the KD embryo.

A notable size difference between KD and SD embryos was also observed at E17.5, as shown in Figure 4. However, unlike at E13.5, at this time point the KD embryo was found to be *smaller*than its SD counterpart. The average embryo image constructed using all (20) SD and KD embryos appears in Figure 4A. Note that the high resolution was maintained during image registration of embryos.

**Figure 5:**
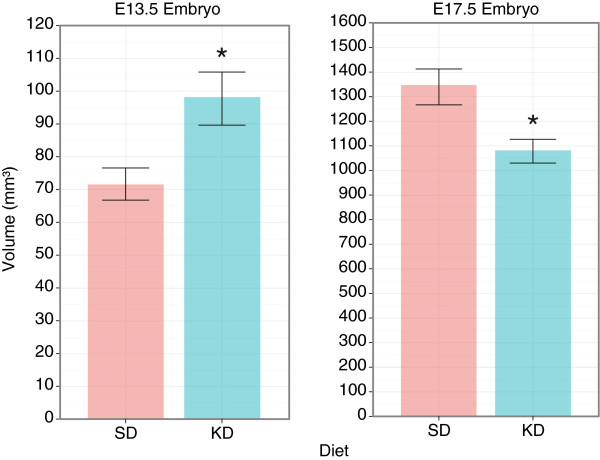
**Embryo volume (Mean±Stdv) in *mm***^**3 **^**at E13.5 (N**_***SD***_** = 27, N**_***KD***_** = 32) and E17.5 (N**_***SD***_** = N**_***KD ***_** = 10).** (*) indicates p < 0.05.

An organ-based analysis on the average embryos revealed that at E13.5 the KD embryo was *larger* than the SD counterpart by 37% (p < 0.05), while at E17.5 the KD embryo was *smaller* than the SD one by 20% (p < 0.05). These differences are depicted in the graph in Figure 5, which shows the volume in *mm*^3^.

The same organ-based analysis was carried out on selected internal organs: the brain, heart, and liver. This analysis allowed us to compute the organ volume as a percentage of the embryo volume. These calculations revealed that the KD brain occupied a smaller percentage of the embryo volume at E13.5, as compared with the SD brain (p < 0.05), but a larger percentage volume at E17.5 (p < 0.05). This result suggests that the growth of the KD brain is shifted towards late pregnancy compared with the growth of the SD brain, but at that point it also exceeds the SD brain growth.

**Figure 6:**
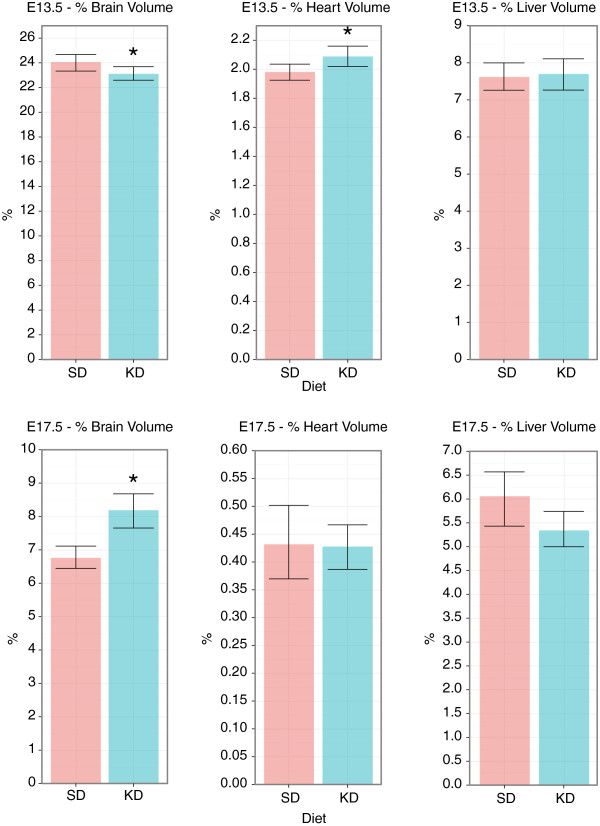
**Percentage volume (Mean±Stdv) occupied by the brain, heart, and liver at E13.5 (N**_***SD***_** = 27, N**_***KD***_** = 32) and E17.5 (N**_***SD***_** = N**_***KD***_** = 10).** (*) indicates p < 0.05.

Unlike the brain, the KD heart was found to occupy a larger percentage volume compared with the SD heart at E13.5 (p < 0.05), but an equivalent percentage volume at E17.5 (p > 0.05). Hence, the dis-proportionality between the KD heart and embryo volumes at E13.5 no longer existed at E17.5, suggesting the KD heart growth exceeds that of the SD heart during organogenesis but not afterwards. The KD liver was found to occupy the same percentage volume of the embryo at both time-points (p > 0.05), indicating the KD liver volume grew proportionally to the embryo volume both during as well as post organogenesis. The percentage organ volume for the different organs is summarized in Figure 6.

### Deformation-based analysis

**Figure 7:**
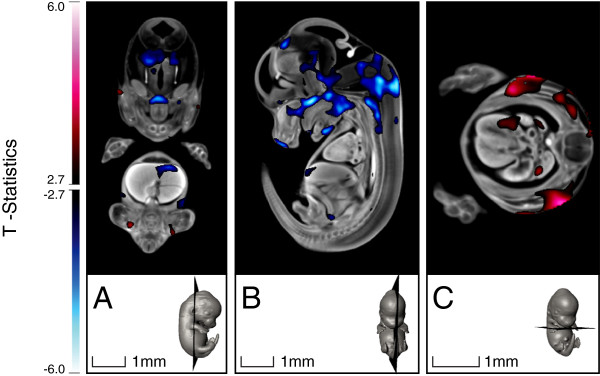
**T-statistics map overlaid on top of the registered E13.5 embryo image, highlighting voxels in the KD embryos that had statistically different deformations compared with those of the SD embryos (FDR ≤ 10%).** Red regions are statistically larger in the KD embryo, whereas blue regions are statistically smaller in the KD compared with the SD embryo.

More localized anatomical changes were detected via a deformation-based analysis conducted on the deformation maps. This analysis at E13.5 revealed that, when compared with the SD embryo, the KD embryo had a decreased relative volume within the pharynx, the cervical spinal cord, and cerebral thalamus region, as well as in the upper hepatic region. Increased relative volume was detected in the lateral wall of the left ventricle of the heart as well as in the skeletal muscle, as shown in Figure 7. Large variations in the position of the tail and limbs with respect to the embryo body existed in the images, arising from embryo positioning within the agar. This could have caused errors in the image registration of the tail and limbs and, as such, they were excluded from the statistical analysis.

**Figure 8:**
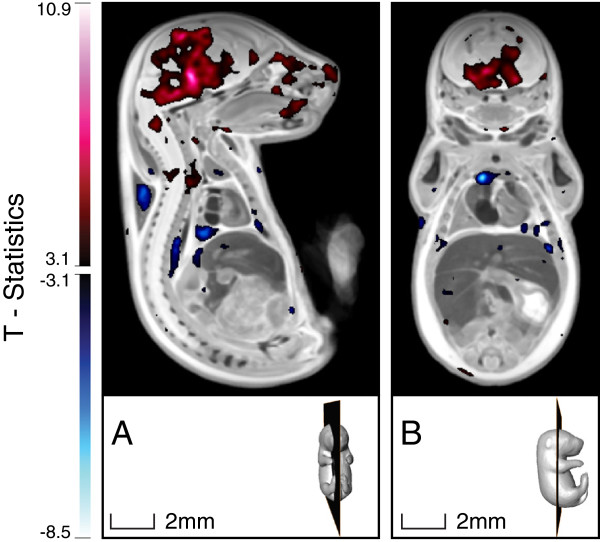
**T-statistics map overlaid on top of the registered E17.5 embryo image, highlighting voxels in the KD embryo that had statistically different deformations compared with those of the SD embryo (FDR ≤ 10%).** Red regions are statistically larger in the KD embryo, whereas blue regions are statistically smaller in the KD compared with the SD embryo.

A deformation-based analysis at E17.5 indicated that the KD mouse had an increase in volume in several brain regions and in the cervical spine, and a decrease in volume in the right lobe of the thymus, as shown in Figure 8.

**Figure 9:**
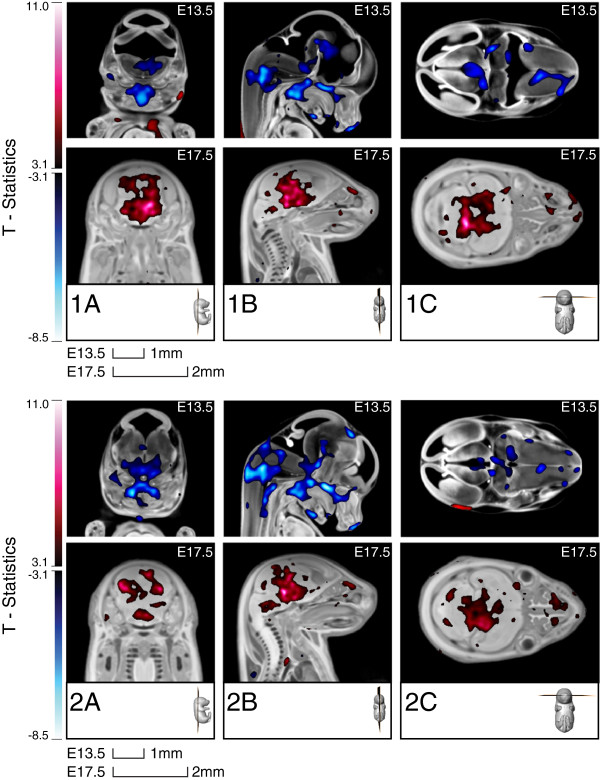
**E13.5 and E17.5 brain regions that are statistically different in the KD compared with the SD embryos.** The top and bottom panels show several selected cross-sections centered around the slices depicted in the 3D rendering. T-statistics color-bar corresponds to a FDR ≤ 10%.

A more detailed examination of the KD brain at the two time points revealed that the mid-brain and pons were smaller in relative volume at E13.5, but larger in relative volume at E17.5 (Figure 9). Additionally, the hypothalamus was smaller in relative volume at E13.5, and the thalamus was enlarged at E17.5, whereas these regions were not statistically different at the other time-points. Overall, this result is consistent with differences in overall brain size found using organ-based analysis. However, it further reveals that specific brain regions show altered growth due to the maternal consumption of KD.

## Discussion

Our study first reveals the organ sizes of the mouse embryo at several time-points during development. These values, calculated in percentage volume, are summarized in Table 2, and are compared with the corresponding organ weight as percent of body weight for the adult 16 week-old mouse [21].

**Table 2:** Organ-to-body ratio in the SD mouse embryo compared with the adult mouse

	**E13.5**	**E17.5**	**16 wks postnatally**
Brain	24.06±0.67% vol.	6.76±0.32% vol.	1.40±0.15% wt.
Heart	1.98±0.06% vol.	0.43±0.07% vol.	0.53±0.07% wt.
Liver	7.52±0.36% vol.	6.13±0.54% vol.	4.87±0.34% wt.

Percentage organ volume of the SD mouse embryo (refer to Figure 6) compared with the percentage organ weight of the adult mouse (averaged across 11 inbred strains) [20] (Mean ±Stdv).

The data reveal that the brain, heart and liver occupy a relatively large percentage of the mouse embryo’s volume at the end of organ development (E13.5). While the percentage volume of the liver decreases slowly towards parturition (E17.5), the corresponding percentages of the brain and heart decrease much more drastically. A comparison with percentage organ weight of an adult 16-week-old mouse reveals that the organ-to-body proportionality continues to decrease significantly post-natally.

Our study also compares how organ growth in the mouse is altered by maternal consumption of a ketogenic diet during pregnancy. Results show an overall increase in volume of the KD embryo at E13.5, but a significant decrease at E17.5, compared with the SD counterpart. At these time points, the KD heart and brain occupied a different percentage volume compared with the SD organs. Additionally, differences revealed by deformation analysis within internal organs were also observed at the two time points, particularly in the pharynx, cervical spinal cord, hypothalamus, thalamus, thymus, mid-brain, pons, left ventricle and in several regions in the liver.

Maternal consumption of KD prior to gestation was found to decrease serum triglycerides in the mouse. This result is similar to that reported by others utilizing the mouse [22]. In contrast, the KD was found to increase triglycerides in the human [23], indicating that the rate of utilization of fat-stores for energy may be different between the two species. However, this needs to be further investigated.

Serum glucose was also found to be reduced, while cholesterol and ketone concentrations were found to be significantly elevated in the KD dams prior to gestation. These changes are as expected during a stable state of ketosis, in which fat intake - including cholesterol intake - is consistently high, and carbohydrate intake is consistently low [2,24,25]. All of these metabolites continued to change in the KD dams during pregnancy, with maternal glucose dramatically dropping, while ketone and triglycerides - drastically increasing between E13.5 and E17.5, towards the end of pregnancy. This trend suggests an increase in nutrient demand by the mature fetus, driving maternal metabolism towards high ketosis. Such a state of high ketosis increases the probability of ketoacidosis in the KD dams since any further drop in glucose could trigger more rapid gluconeogenesis, elevating ketones to dangerously high levels, and thus, preventing the utilization of the already-elevated serum ketones. While ketoacidosis was not observed in any of the KD dams during pregnancy, the high ketosis, and particularly the dramatically low maternal blood glucose, could have been the cause of the altered growth exhibited at E17.5. This deleterious effect could have impacted those organs which preferentially use glucose as a source of energy.

While this ketone (*β*−hydroxybutyrate) can lead to a risky metabolic state, it is in fact a natural product of fat metabolism [26], which also serves as a metabolic fuel [27,28]. Since maternal ketones easily cross the placenta [29,30], they should be similarly utilized by the fetus to satisfy its energy requirement during development [27,28]. If that were indeed the case, then one could naively predict that during a stable, prolonged maternal ketosis, fetal growth would be unaltered, and remain equivalent to that during maternal glycolysis. This, however, neglects to consider the inherent differences between glycolysis and ketosis. While ketones *can* satisfy energetic requirements, their efficiency as a metabolic fuel is different than that of glucose. In fact, ketones’ efficiency, as measured by oxygen consumption, has been shown to be 25% greater than that of glucose, indicating a significantly greater energy production during ATP hydrolysis [31-33]. Such increased efficiency may then be counteracted by the inhibitory effect of ketones on *de novo* pyrimidine synthesis, which can slow the rate of cellular growth, as shown in Koehler et al’s *in vitro* studies (described in [24]). The net result of these opposing actions on embryonic development and fetal growth would then depend on the exact nutrient and energetic demand at any particular developmental phase. Specifically, a positive net effect could explain the increase in embryonic volume, observed at E13.5, yet, a negative – or inhibitory – net effect could, in turn, explain the decrease in embryonic volume, observed at E17.5. Internal organs are potentially also subjected to the same counteracting forces, resulting in altered timing of growth spurts, which could explain the altered KD brain and heart percentage volume at E13.5 and E17.5.

Different effects of KD on organ growth may be explained by organ-specific preference for a particular metabolic fuel. Under non-ketotic conditions, the fetal myocardium has been shown to prefer fatty acids, glucose and lactate over ketones as its major energy substrates [34,35]. This preference, as measured by substrate utilization rate, was found to primarily depend on relative substrate availability (concentration) [34]. When ketosis was induced by an over-night fast, the neonatal lamb myocardium increased its *βOHB* oxidation in response to the increase in ketone supply. A similar increase in *βOHB* oxidation was reported in the brain of the swine fetus following maternal ketosis [36]. In the fetal pig, the brain was also found to increase in weight, protein content and cell size following maternal ketosis [37]. Yet, even independently of maternal ketosis, the developing mouse brain and spinal cord appear to preferentially utilize ketone bodies as an energy source, and as precursors of amino-acid and lipid synthesis [38,39]. In particular, the rat brain – specifically, the striatum and cortex – were found to have a significantly higher *βOHB* tissue concentration in late gestation and early postnatal period than at adulthood [40]. Unlike the heart and brain, the fetal liver has been reported to only increase its rate of lipogenesis, but *not* its rate of *βOHB* oxidation (i.e. degradation), during maternal ketosis [36]. The potential implication of this is a more rapid triglyceride synthesis, but with a preference for fat deposition in the liver, that could result in an increase in hepatic volume. This, however, was not observed in our KD mice, suggesting the effect of hepatic fat deposition on hepatic volume is insignificant.

Ketone bodies in maternal circulation cross the placenta so that they are available as an energy substrate for fetal use throughout gestation [30]. However, such availability depends on the ketone body carriers, known as Monocarboxylate Transporters (MCTs) [41,42]. Studies using non-ketogenic gestational diet revealed that the expression of MCT in the placenta decreases at the end of gestation, while the expression of the glucose transporter GLUT1 remains consistently high throughout gestation [29]. This observation suggests that assuming ketolytic enzymes are available to facilitate ketone oxidation [43], then the fetal mouse utilizes both glucose and ketone in early and mid gestation. However, it preferentially utilizes glucose towards the end of gestation, when rapid physiological growth is occuring. In the case of the fetal brain, ketone utilization primarily depends on its concentration in the blood [39]. Hence, prolonged fasting or consumption of a high-fat diet, such as a ketogenic diet, increases the blood-brain barrier’s permeability to ketones [39,44,45].

Our observation that the KD embryonic volume as well as myocardium and hepatic percentage volumes were larger than the respective SD ones at E13.5 but not at E17.5 are consistent with the above literature on *βOHB* oxidation and supply, as modulated by MCT expression. The observation of a localized increase in mid-brain volume in the KD fetus at E17.5, is also in agreement with the studies indicating a strong preference for *βOHB* as an energy substrate towards the end of gestation [40], as well as with the observation of the increase in fetal brain weight [37]. The high PUFA contents in the KD may result in PUFA accumulation in the brain at the end of gestation, and explain the increased brain volume. This is in agreement with observations by Soares et al. (2009) [46]. The increase in skeletal muscle volume observed at E13.5 may be indicative of increased lipogenesis, while the localized differences indicated by the deformation analysis within internal organs could arise from inhomogeneous energy supply to those sub-regions, or their preference for a different metabolic fuel during their growth.

Another possible cause of the observed differences between the KD and SD embryos may be the inherent difference in macronutrient and micronutrient contents of the respective diets. For example, the KD is high in dietary trans-fatty acids. Studies have indicated a speculative correlation between such high gestational intake of trans-fatty acids and compromised fetal growth and development [47]. In our study, the exact contents of trans fatty-acids was not measured, but may contribute to the observed differences between the KD and SD embryos. Hence, we do not exclude the possibility that some of the observed alterations may be attributed to the differences in micro- and macronutrient intake.

Overall the differences revealed by deformation analysis suggest a deviation from the normal organ development at the end of embryogenesis, which could lead to altered organ function in postnatal life. Specifically, the decrease in relative volume of the right lobe of the thymus, as well as the increase in relative volume in the thalamus, mid-brain, and pons at E17.5 may be indicative of functional changes of those organs. The exact functional effects remain to be elucidated by post-natal studies. Due to the genetic similarity between the mouse and the human, our results could also shed some light on the potential growth effects on the human fetus, if exposed to a similar gestational ketogenic diet.

## Conclusion

To elucidate some of the possible consequences of maternal KD consumption to fetal growth, we imaged mouse embryos whose mothers were either on a Standard Diet (SD) or a Ketogenic Diet (KD) during gestation. Our data from the SD embryos were compared with currently available literature on the adult mouse, and reveals a dramatic change in organ-to-body ratio between E13.5, E17.5, and adulthood. A comparison between the SD and KD embryos further indicate marked alterations in overall embryonic growth, as measured by embryo volume and organ percentage volume, as well as differences revealed by deformation analysis within organs; the brain, spinal cord, pharynx, heart, thymus, and liver. These growth changes may be a result of embryonic and organ energy substrate preference, which are modulated by their concentration in maternal circulation, as well as their transport across the placenta at the various gestational time-points. The drastic drop in maternal blood glucose along with the increase in blood ketone towards the end of gestation suggest ketones were the primary energy substrate available for the developing fetus during its rapid growth phase. As such, ketones could have been responsible for the altered KD growth at both time-points, as well as for the relatively hindered KD growth observed at E17.5 compared with its growth at E13.5. Overall, these growth alterations could be indicative of underlying functional, physiological alterations which could become apparent in postnatal life.

## Abbreviations

3D: Three dimensional; *βOHB*: *β* − *hydroxybutyrate*; E: Embryonic day; FDR: False Discovery Rate; Gd: Gadolinium; KD: Ketogenic diet; MCTs: Monocarboxylate transporters; MRI: Magnetic resonance imaging; OPT: Optical Projection Tomography.

## Competing interests

The authors declare that they have no competing interests.

## Authors’ contributions

DS and SLA created the study protocol. DS and MDW conducted the study. DS collected the results. DS and MVE analyzed the data. DS and MH supervised the study and wrote the manuscript. All authors read and approved the final manuscript.

## Pre-publication history

The pre-publication history for this paper can be accessed here:

http://www.biomedcentral.com/1471-2393/13/109/prepub
